# The sero-epidemiology of human papillomavirus among Caucasian transplant recipients in the UK

**DOI:** 10.1186/1750-9378-4-13

**Published:** 2009-09-14

**Authors:** Delphine Casabonne, Tim Waterboer, Kristina M Michael, Michael Pawlita, Aoife Lally, Liza Mitchell, Beata Imko-Walczuk, Fenella Wojnarowska, Robert Newton, Charlotte Proby, Catherine Harwood

**Affiliations:** 1 Cancer Epidemiology Unit, Richard Doll Building, University of Oxford, Old Road Campus, Roosevelt Drive, Headington, Oxford, OX3 7LF, UK; 2 Infection and Cancer Program (F020), German Cancer Research Center (DKFZ), Im Neuenheimer Feld 280, 69120 Heidelberg, Germany; 3 Department of dermatology, Churchill Hospital, Oxford Radcliffe Hospitals, Oxford, OX3 7LJ, UK; 4 Centre for Cutaneous Research, Institute of Cell and Molecular Science, Barts and the London School of Medicine and Dentistry, Queen Mary, University of London, London E1 2AT, UK; 5 Clinical Department of Plastic Surgery, Medical Academy of Gdańsk, Poland, ul. Dębinki 7, 80-952 Gdańsk; 6 Epidemiology & Genetics Unit, Department of Health Sciences, University of York, Area 3, Seebohm Rowntree Building, Heslington, York, YO10 5DD, UK; 7 Division of Surgery and Oncology, Ninewells Hospital & Medical School, University of Dundee, Dundee DD1 9SY, UK

## Abstract

**Background:**

Despite intensive study of high-risk mucosal human papillomaviruses (HPV), little is known of the epidemiology of cutaneous HPV. As part of a study of cutaneous squamous cell carcinoma and HPV among organ transplant recipients (OTR) from London and Oxford, we investigated the seroprevalence and risk factors for 34 HPV types (detected using Luminex technology) among 425 Caucasian OTR without skin cancer.

**Results:**

Overall, 86% of participants were seropositive to at least one HPV: 41% to mucosal alpha types, 33% to cutaneous alpha types, 57% to alpha types, 56% to beta, 47% to gamma types and 45% to other types (nu, mu, HPV101 and 103). In both centres, the most common types were HPV6 (33% and 26% for London and Oxford respectively), HPV8 (24% and 18%), HPV15 (26% and 29%), HPV17 (25% and 21%), HPV38 (23% and 21%), HPV49 (19% and 21%), HPV4 (27% and 23%), HPV65 (30% and 25%), HPV95 (22% and 20%), HPV1 (33% and 24%) and HPV63 (28% and 17%). The seroprevalence of 8 HPV types differed significantly (P < 0.05) between London and Oxford. Those individuals seropositive to multiple types of one genus were more likely to be seroreactive to multiple types of another genus. As expected, antibodies against mucosal alphaHPV types were more frequent in younger patients and among women. Sunbed use and sunbathing was associated with seropositivity to multiple gammaHPV (P-trend = 0.007) and self-history of abnormal smear was related to seroactivity to multiple betaHPV (P = 0.01). Skin type and other self reported markers of exposure to ultraviolet radiation were not consistently associated with any HPV types. No other distinguishing epidemiological features of transplant recipients with antibodies against single or multiple HPV types were identified.

**Conclusion:**

Findings for mucosal HPV types were in line with results from previous studies. We observed differences in HPV seroprevalence between organ transplant recipients from two geographically close centres but no clear risk factor was found associated with cutaneous HPV seropositivity among organ transplant recipients. These findings have implications for interpretation of future seroepidemiology studies addressing the association between HPV and cutaneous SCC in OTR populations.

## Background

Papillomaviruses are small circular double-stranded DNA viruses of around 8 kb. To date, at least one hundred and eighteen papillomaviruses have been completely described of which approximately 100 are human and the remainder are animal types. Based on DNA analysis, the HPV phylogenetic tree is composed of 5 genera (alpha, beta, gamma, mu and nu papillomaviruses) which, in turn, are grouped into species and subdivided into types [1].

Human papillomaviruses infect either cutaneous or mucosal epithelium. High-risk mucosal HPV types (e.g. 16, 18, 31 and 33) are causative for cancers of the uterine cervix [2], but other types are responsible for benign cutaneous viral warts [3]. For instance, common warts seen on the skin of arm, hand and leg are often associated with HPV 1, 2, 4, 7 and 57; flat warts are usually caused by HPV 3, 10 and sometimes 2 and are more commonly observed in immunosuppressed patients or patients with a rare inherited skin disease, epidermodysplasia verruciformis. Genital warts (condyloma acuminata), oral warts and low-grade cervical squamous intraepithelial lesions are mainly caused by HPV 6 and 11. In addition, HPV are ubiquitous viruses which are also detected in healthy skin and hair follicles [4] and betaHPV types might be involved in the pathogenesis of cutaneous SCC in patients with a rare skin disease, epidermodysplasia verruciformis [5].

The natural history of high-risk HPV types in relation to cervical cancer has been studied intensively [2] but few data are available on the seroprevalence and risk factors associated with the other HPV types [6], especially cutaneous HPV types. Organ transplant recipients (OTR) are an important high risk population to study since there is an up to 100-fold increased risk of SCC in these patients compared to the general population [7,8]. While the role of betaHPV in the development of SCC is still unclear, a better understanding of the epidemiology of HPV is also important for future studies on SCC. Here, we investigate the seroprevalence and risk factors for 34 HPV types detected using Luminex technology among 425 OTR Caucasian without skin cancer.

## Methods

### Study population

The present study was conducted as part of research examining the relationship between antibodies against the major capsid protein L1 of 34 HPV types and cutaneous squamous cell carcinoma among OTR. More information on the study design and data collection can be found elsewhere [9]. Overall the response rates were 96% and 82% in London and Oxford respectively and all eligible patients have had an equal opportunity to enter the study. All organ transplant recipients from Oxford Radcliffe Hospitals and from Barts and London NHS Trust were invited to participate in the study and were recruited between October 2002 and August 2006. Patients underwent transplantation between 1964 and 2005 in Oxford and between 1972 and 2006 in London. In London, all patients have access to a dedicated dermatology clinic following their usual visit to the transplant centre, are seen routinely within 6-12 months of transplant and undergo routine dermatological examinations thereafter at which all benign and malignant lesions are recorded and treated if necessary. For the present study, patients were recruited at routine clinic visits and completed a questionnaire delivered by a specialist nurse and were examined by a dermatologist. In Oxford, patients are referred to a dermatologist if a suspicious skin lesion is present, but are not otherwise under routine surveillance. Therefore, OTR attending the Oxford Transplant Centre were invited by mail to take part in the study and to complete a questionnaire. At the next clinic visit, this questionnaire was checked and finalised by a dermatologist who also conducted an examination of the participants' skin, recording all benign and malignant cutaneous lesions. Treatments were initiated where indicated and educational information relating to the risks of skin cancer in OTR was also provided. In both centres, a blood sample was taken and serum, buffy coat and red blood cells were separated, aliquoted and frozen at -80°Celsius. In total, 425 OTR patients without skin cancer were recruited in the 2 centres (243 from London and 182 from Oxford).

### Questionnaire

The same questionnaire was used in both centres to collect information on (i) social and demographic details (age, sex, height, weight, ethnicity, marital status, educational level, area of residence, country of birth) (ii) smoking and alcohol history; (iii) medical history (skin and/or other cancers, psoriasis); (iv) exposure to ultraviolet (UV) radiation (outdoor occupation and hobbies, sun exposure before and after transplantation, sun exposure currently, number of moles and freckles before and after transplantation, history of sunburn in childhood, protective measures against UV radiation, time spent abroad); (v) history of HPV-related viral infection (cutaneous and genital warts and history of abnormal smear in women); (vi) transplantation and dialysis (number of transplantations, dates, type of dialysis, time spent on dialysis before and after transplantation, primary diagnosis); (vii) gynaecological and reproductive history for women (age at menopause, number of pregnancies, use of hormonal contraception, hormone replacement therapy, surgical removal of uterus). Since it is not clear how cutaneous HPV are transmitted, the questionnaire also included some questions on possible risk factors for infection (e.g. shared bedroom or bed as a child, number of siblings and number in household, as surrogates for crowding and proximity). No information on HLA or other infections was collected. Current immunosuppressive treatment at recruitment was documented, but a detailed retrospective history on specific or combined immunosuppressive treatments was not recorded. All information on transplantation, medications and skin cancers was cross-checked against information held in the renal-centre database and medical records.

### Ethical approval

The study in London was approved by the East London and City Health Authority Research Ethics Committee and in Oxford by the Mid- and South Buckinghamshire Local Research Ethics Committee.

### HPV multiplex serology

HPV antibody detection was performed by multiplex serology, an antibody detection method that is based on a glutathione *S*-transferase (GST) capture enzyme-linked immunosorbent assay, as previously described [10,11] in combination with fluorescent bead technology [12,13]. All antigens were expressed in *E. coli *as double fusion of full-length viral proteins with a N-terminal GST domain and a C-terminal peptide consisting of the last 11 amino acids from the large T antigen of simian virus 40 [10]. The expression constructs for the full length L1 proteins of all HPV types analyzed here (mucosal alpha: 6, 13 and 16; cutaneous alpha: 2, 3, 7 and 27; beta: 5, 8, 9, 15, 17, 20, 23, 24, 36, 38, 49, 75, 76, 92, 93, 96; gamma: 4, 65, 95, 48, 50, 60; nu: 41; mu: 1, other types: 101 and 103) are described in detail elsewhere [6,11,14]. Glutathione-casein was coupled to internally fluorescence-labeled polystyrene beads (Luminex, Austin, TX), and fusion proteins were affinity-purified on the beads directly in a one-step procedure. Beads with GST and the C-terminal peptide alone were prepared for background determination. Binding of the antigens (i.e. the GST fusion proteins) to various bead sets was verified with a monoclonal antibody against the common C-terminal peptide [10]. The differently labeled bead sets carrying different antigens were mixed and incubated in 96-well plates with human plasma diluted 1:100 in blocking buffer, as described previously [13]. Antibodies bound to the beads via the viral antigens were then stained with biotinylated anti-human immunoglobulin and fluorescent reporter conjugate streptavidin-R-phycoerythrin. Antibodies bound to antigens on beads were quantified via the reporter fluorescence in the Luminex analyzer, which also identified the internal bead colour and thus the antigen carried by the bead. Antibody quantity was determined as the median R-phycoerythrin fluorescence intensity (MFI) from at least 100 beads of the same internal colour after subtraction of background reactivity (GST and C-terminal peptide alone).

The assay reproducibility was high (R^2 ^= 0.97) [12,15]. More information on quality control has been described elswewhere [15]. For all HPV types but HPV6 analyzed here, MFI cut-offs to define seropositivity for all antigens were set to 200 MFI as described and discussed previously [6,14]. To reduce the influence of borderline seropositive sera, a stringent (doubled) cut-off of 400 MFI was applied to HPV6. In our previous analysis [14], data analysis using geometric mean MFI values instead of cut-off values did not materially change the results.

### Statistical methods

To assess the relationship between seropositivity to a single HPV type and various risk factors measured by questionnaire, conditional (on centre) logistic regression adjusted for sex, age at recruitment (<45, 45-59, ≥ 60 years) and time since transplantation (<5 years, 5 to 9 years, ≥ 10 years) was applied. To examine the association between the total number of HPV seropositivity (count) and risk factors, negative binomial regression adjusted for the same factors and centre was preferred since over-dispersion was observed when Poisson models were fitted (likelihood ratio test for the null hypothesis of no overdispersion was rejected with P < 0.001). Where results are presented in the form of plots, black circles indicate the point estimates and horizontal lines represent 95% confidence intervals (CI). Skin type was defined using Fitzpatrick classification scale as follows (I) never tans, always burns, (II) rarely tans, usually burns, (III) usually tans, can burn and (IV) always tans, rarely burns.

To deal with multiple significant tests the level of statistical significance was set to 1% and when a sufficient number of patients was available, agreement of results across centre was used to detect genuine associations. Missing value categories were added to adjustment variables with incomplete information in order to retain all the observations in the analyses. Likelihood ratio tests were used to assess heterogeneity tests. All P-values are two-sided. Statistical analyses were carried out using STATA 9 (StataCorp, 2005).

## Results

### Participants

In total, 425 Caucasian OTR (5 patients [0.8%] from London with a solid organ graft other than kidney) with a blood sample and a completed questionnaire, but without skin cancer were available for analyses. Table 1 shows the distribution of OTR by sex, time since transplantation and age at recruitment for each centre. There was no difference at the 5% level of significance in distribution between the two centres in terms of these factors.

**Table 1:** Descriptive statistics for age at recruitment, sex and time since transplantation among Caucasian transplant patients by centres (N = 425)

	**Oxford**	**London**	**Total**
	**N = 182**	**N = 243**	**N = 425**
	**no (%)**	**no (%)**	**no (%)**
**Sex**			
male	110 (60)	150 (62)	260 (61)
female	72 (40)	93 (38)	165 (39)
***P-het.***		***0.8*^1^**	
**age at recruitment (years)**			
<45	80 (44)	107 (44)	187 (44)
45-59	64 (35)	94 (39)	158 (37)
60 or more	38 (21)	42 (17)	80 (19)
***P-trend***		***0.6^1^***	
**time since transplantation (years)**			
<5	70 (38)	80 (33)	150 (35)
5 to 9	52 (29)	61 (25)	113 (27)
10 or more	60 (33)	102 (42)	162 (38)
***P-trend***		***0.08^1^***	

N: Total number; no: number.

^1 ^P-values between Oxford and London are based on logistic regression adjusted

for the other factors.

### Prevalence and ubiquity of HPV

Table 2 shows the HPV seroprevalence by genus and summarises the mean number of serotypes. Those individuals seropositive to multiple types of one genus were more likely to be seroreactive to multiple types of another genus. Eighty-six percent of patients were seropositive to at least one HPV type with 41%, 33%, 56%, 47% and 55% of patients being seroactive to any alpha mucosal, alpha cutaneous, beta, gamma and other HPV types respectively. There was no difference in terms of age at recruitment, time since transplantation or sex between HPV seronegative patients to all types and those being seropositive to at least one HPV type (Table 3). Of the 425 Caucasians OTR, 237 (56%) were seropositive to any beta types, 205 patients (48%) were seropositive to HPV8, 9, 15, 17, 38 and/or 49 and only 32 patients (8%) were seropositive to any of the other 10 betaHPV types. Regarding the gamma types, 200 patients (47%) were seropositive to any gammaHPV types, 178 (41%) were seropositive to HPV4, 65 and/or 95 (species 1) and 22 patients (5%) were seropositive to any of the three other types (data not shown).

**Table 2:** Human papillomavirus seroprevalence by genus and mean number (SD) of HPV seropositivty per Caucasian transplant patient across genus (N = 425)

			**Mean (SD) number of HPV seropositivity per patient and by genus**			
			
			**alpha**	**beta**	**gamma**	**other**
		**Number of HPV seropositive patients (%)** **N = 425**	***(7 types)***	***(16 types)***	***(6 types)***	***(5 types)***
**Seropositivity to:**						
**Any types**		366 (86)	1.0 (1.3)	2.4 (3.7)	1.0 (1.4)	0.8 (1.1)
						
**alpha mucosal**						
	**0**	250 (59)		1.9 (3.3)	0.8 (1.2)	0.6 (0.9)
	**1**	127 (30)		2.2 (3.4)	1.1 (1.5)	0.8 (1.0)
	≥ **2**	48 (11)		5.3 (5.1)	2.0 (1.7)	1.4 (1.4)
**alpha cutaneous**						
	**0**	285 (67)		1.6 (2.8)	0.7 (1.1)	0.6 (0.9)
	**1**	92 (22)		2.6 (3.6)	1.2 (1.5)	0.7 (1.0)
	≥ **2**	48 (11)		6.8 (5.5)	2.6 (1.8)	1.8 (1.6)
**beta**						
	**0**	188 (44)	0.6 (0.8)		0.4 (0.8)	0.4 (0.7)
	**1**	76 (18)	0.8 (0.9)		0.5 (0.7)	0.5 (0.8)
	**2/3**	76 (18)	1.3 (1.5)		1.2 (1.4)	0.6 (0.9)
	≥ **4**	85 (20)	2.0 (1.8)		2.7 (1.7)	1.8 (1.4)
**gamma**						
	**0**	225 (53)	0.7 (0.9)	0.8 (1.5)		0.4 (0.6)
	**1**	92 (22)	1.0 (1.2)	1.7 (2.2)		0.9 (1.0)
	≥ **2**	108 (15)	1.9 (1.7)	6.1 (5.2)		1.5 (1.4)
**other types**						
	**0**	233 (55)	0.8 (1.1)	1.2 (2.2)	0.6 (1.0)	
	**1**	107 (25)	1.0 (1.1)	2.3 (3.5)	1.0 (1.4)	
	≥ **2**	85 (20)	1.8 (1.8)	5.6 (5.2)	2.3 (1.8)	

HPV: human papillomavirus; N: number; SD: standard deviation; Other types (nu, mu, HPV101 and HPV103). Using negative binomial regression adjusted for age at recruitment, sex, time since transplantation and center, all P-trend obtained were less than or equal to 0.001

**Table 3:** Age, sex and time since transplantation distribution in HPV seropositives compared to seronegatives among Caucasian transplant patients without skin cancer from London and Oxford (N = 425)

	**Seronegative to all HPV types**	**Seropositive to at least one HPV type**	**total**
	**N = 59**	**N = 366**	**N = 425**
**Sex**			
male	25 (42)	140 (38)	**165 (39)**
female	34 (58)	226 (62)	**260 (61)**
*P-het.*		***0.5***	
**age at recruitment**			
<45	24 (41)	163 (45)	**187 (44)**
45 to 59	20 (34)	138 (38)	**158 (37)**
≥ 60	15 (25)	65 (18)	**80 (19)**
*P-trend*		***0.3***	
**time since transplantation**			
<5	24 (41)	126 (34)	**150 (35)**
5 to 9	11 (19)	102 (28)	**113 (27)**
≥ 10	24 (41)	138 (38)	**162 (38)**
*P-trend*		***1.0***	

N: number; HPV: Human papillomavirus; het: heterogeneity. P-value using conditional

(on centre) logistic regression adjusted for each others.

### Risk factors associated with single HPV seropositivity among Caucasian OTR

Additional file 1 shows seroprevalence of the 34 HPV types by centre, sex, age at recruitment, time since transplantation and skin type. The prevalence of 8/34 HPV types differed significantly (P ≤ 0.05) between centres. In both centres, highest seroprevalence was observed for HPV6 (33% and 26% for London and Oxford respectively), HPV8 (24% and 18%), HPV15 (26% and 29%), HPV17 (25% and 21%), HPV38 (23% and 21%), HPV49 (19% and 21%), HPV4 (27% and 23%), HPV65 (30% and 25%), HPV95 (22% and 20%), HPV1 (33% and 24%) and HPV63 (28% and 17%). Seroprevalence was statistically significantly higher at the 1% level in London compared to Oxford for 3 cutaneous types; HPV 27 (21% versus 12% respectively), HPV 63 (28% versus 17%) and HPV 101 (10% versus 3%), and for 1 mucosal type, HPV 13 (13% versus 5%). As expected, higher HPV seroprevalence was observed in women for HPV16 (25% in female versus 10% in male) and seroprevalence for mucosal HPV types (16, 6 and 13) decreased with increasing age (P-trend < 0.01). The same associations were found when a larger number of age categories was used (<35, 35-39, 40-44, 45-49, 50-54, 55-59, ≥ 60) (data not shown). Apart from an increase in seroprevalence of HPV4 with time since transplantation (P-trend = 0.01) and a decrease in seroprevalence of HPV65 with increasing age (P-trend < 0.001), no clear association was found with time since transplantation for any of the other HPV types examined. Similarly, no association was found between HPV seroprevalence and skin phototype.

Regarding the other risk factors, patients who were seropositive to HPV6 were more likely to be ex- or current smokers, to have had more children (P-trend < 0.001) and to have had a history of genital warts and/or abnormal cervical smear. A self-reported history of psoriasis (n = 23 patients) was not associated with any of the 34 HPV types examined. Among women, an association was found between history of abnormal cervical smear and presence of antibodies against many betaHPV types (HPV 15, 17, 38, 76 and 92). However, across the 2 centres, only 28 women had such histories and results could not be examined by individual centre (data not shown). No other distinguishing epidemiological features of transplant recipients with antibodies against any of the 34 HPV types examined were identified (data not shown).

### Risk factors associated with multiple HPV seropositivity among Caucasian OTR

**Figure 1:**
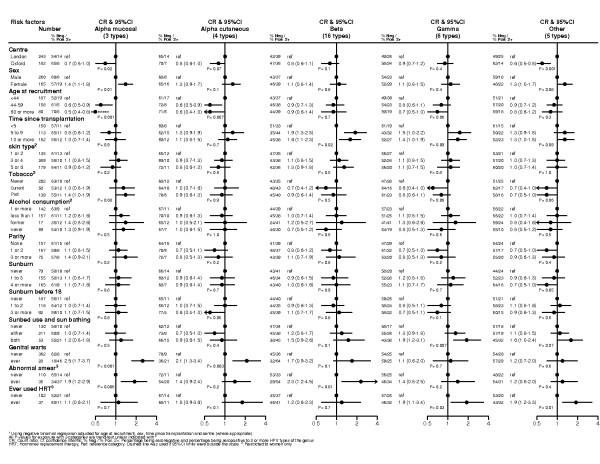
**Risk factors associated with multiple HPV seropositivity among Caucasian transplant patients without skin cancer from London and Oxford (N = 425)**.

Figure 1 is a graphical representation of selected risk factors associated with the presence of antibodies against multiple HPV types by genus. Multiple HPV seropositivity was more frequent in patients from London as compared to Oxford for alpha mucosal types and for other types (nu, mu and 2 not defined types). Young women, past smokers and female patients reporting a history of having had an abnormal cervical smear test and/or genital warts were more likely to be seroreactive to multiple mucosal HPV types. Women with a self-reported history of abnormal cervical smear were also more likely to be seropositive to more beta HPV types than those without such a history (CR: 2.3; 95% confidence interval: 1.2 to 4.5; P = 0.01) and multiple seropositivity to beta types seemed to be higher in patients with longer time since transplantation (P-trend = 0.02). Ultraviolet radiation exposure history was not associated with seropositivity to a single HPV type but patients who reported using a sunbed and sunbathing were twice as likely to have more gamma serotypes than those who did not (P-trend = 0.007). No other distinguishing epidemiological features of OTR with antibodies against multiple HPV seropositivity were evident.

## Discussion

Little is known of the seroepidemiology of HPV, with the exception of those mucosal types associated with cancer of the uterine cervix [2]. Organ transplant recipients (OTR) are at higher risk of developing skin cancer than the general population [7,8] and HPV has been suggested as having a role in the development of cutaneous squamous cell carcinoma [16,17]. Hence, examining potential risk factors in high risk transplant populations is important to better understand the epidemiology of HPV. We report here on risk factors associated with HPV seropositivity for 3 mucosal and 31 cutaneous HPV types across 5 genera among 425 Caucasian OTR without skin cancer.

Eighty six percent of transplant recipients without skin cancers in this study were seroreactive to at least one HPV type, identical to previous results from a UK study among immunocompetent people [14], illustrating the ubiquity of HPV [4]. We observed differences in HPV seroprevalence between OTR from 2 geographically close centres (Oxford and London) perhaps reflecting the importance of disparity in environmental exposure or even clinical practice. Individuals seropositive to multiple types of one genus were also more likely to be seroreactive to multiple types of another genus. This pattern was also observed in immunocompetent and dialysis patients (Casabonne *et al.*, this issue) suggesting that the association was not due to higher susceptibility to multiple HPV seropositivity of OTR patients following immunosuppressive treatments. Multiple HPV seropositivity could reflect infections with more types due to higher environmental exposure to these viruses, similar modes of transmission or cross-reactivity of the antibodies. Alternatively, it could reflect higher viral load that induced stronger and more frequent antibody responses. Viral load for beta HPV types could be regulated by genetic or other host factors. EV patients, who cannot appropriately control beta HPV replication have high viral loads and strong antibody responses to a broad variety of beta HPV types [18]. Mutations in EVER1 and EVER2 genes have been identified as essential genetic defect in some EV patients [19,20]. In the general population an EVER2 polymorphism has been found positively associated with beta HPV seropositivity as well as with increased risk of SCC [21]. If beta HPV contribute to the pathogenesis of cutaneous SCC it is conceivable that higher viral load could increase the risk.

Findings for mucosal HPV types were in line with results from previous studies [2] and provide internal validation for the multiplex technology used in this study. Associations with time since transplantation and seropositivity to certain beta or gamma types were not linear, but might reflect a decline in antibody production following intensive immunosuppressive treatments in the first years following transplantation. However, this remains speculative and would require prospective HPV serology studies for confirmation. No association was observed between the presence of psoriasis (self-reported) and any of the 34 HPV type examined. Despite the small number of patients, this result is in line with our recent study on beta HPV-DNA and psoriasis [22]. There is limited statistical power to examine associations with all of the HPV types, in part because the prevalence of some is low. Further limitations may arise because details of risk factors were examined using self-reported information.

The largest previous study of HPV seroprevalence in the immunocompetent population reported on age and sex distributions of alpha, beta, gamma, nu and mu HPV types among 1797 German adults and children [6]. Overall, detection of antibodies against nu and mu types was evident in childhood whereas seroprevalence to alpha types was higher in women after puberty; seroprevalence to beta and gamma types was found to increase with age. We did not find an association between age and presence of antibodies against gamma and beta types in our transplant population. Our results in relation to age and betaHPV differed from the German study [6] suggesting that the observed differences might reflect differences between children and adults. Other studies have reported only on beta HPV seroprevalence and risk factors among immunocompetent individuals [16,17,23,24]. Termorshuizen *et al *(2004) reported no association in 313 controls patients between seropositivity to any of 6 beta HPV types (HPV5, 8, 15, 20, 24 or 38) and age, sex, skin type, lifetime sun exposure and painful sunburns at different age periods [23]. Karagas et al (2006) also reported that seropositivity to any of 8 beta types (HPV 5, 8, 9, 15, 20, 24, 36 or 38) in 461 immunocompetent patients without skin cancer did not differ in terms of age, level of education, smoking status, skin phototype and number of sunburns, but noted higher beta seroprevalence in men compared to women [16]. Andersson et al (2008) looked at 434 immunocompetent patients with and without skin cancer (basal and squamous cell carcinoma) and also found no relationship between age, sex, skin type, smoking and previous sunburn and seropositivity to any beta types (HPV 5, 8, 9, 10, 15, 20, 24, 36 and 38) [17]. Only Feltkamp et al (2003) found a statistical significant association between unadjusted seroprevalence of HPV24 and increasing age and male sex in immunocompetent patients [24].

## Conclusion

In summary, findings on the association between various risk factors and mucosal HPV types were an internal validation of the methodological approach used in this study. Significant differences in HPV seroprevalence were identified between study centres. Sunbed use and sunbathing was associated with seropositivity to multiple gammaHPV and, among women, self-history of abnormal smear was related to seroactivity to multiple betaHPV. No other distinguishing epidemiological feature of transplant patients with antibodies against any of the 34 individuals or multiple HPV types examined were identified. Interpretation of future HPV and cancer association studies will require a better understanding of HPV seroepidemiology and further research is now needed to clarify the risk factors and the natural history of these viruses.

## List of abbreviations

HPV: human papilloma virus; OTR: organ transplant recipients; OR: odds ratio; CR: count ratio; CI: confidence interval.

## Competing interests

The authors declare that they have no competing interests.

## Authors' contributions

RN, CP, CH and FW conceived the study. LM, AL, BIW and DC participated in acquisition of all the biological material and data. MP, TW and KM developed the HPV assays and KM analysed the samples. DC analysed the data and drafted the manuscript. All authors read, contributed to and approved the manuscript.

## Supplementary Material

Additional file 1 **Human papillomavirus seroprevalence by centre, sex, age at recruitment, time since transplantation and skin type by each HPV type, among Caucasian transplant patients without skin cancer from London and Oxford (N = 425)**. This file shows seroprevalence of the 34 HPV types by centre, sex, age at recruitment, time since transplantation and skin type. Click here for file
