# Supplementary material for: The sero-epidemiology of human papillomavirus among Caucasian transplant recipients in the UK
Source: Infect Agent Cancer. 2009 Sep 14;4:13. doi: 10.1186/1750-9378-4-13 (PMC2749815; doi:10.1186/1750-9378-4-13)
Supplement: Additional file 1 — Human papillomavirus seroprevalence by centre, sex, age at recruitment, time since transplantation and skin type by each HPV type, among Caucasian transplant patients without skin cancer from London and Oxford (N = 425). This file shows seroprevalence of the 34 HPV types by centre, sex, age at recruitment, time since transplantation and skin type. [file 1750-9378-4-13-S1.doc]

Additional File 1: Human papillomavirus seroprevalence by centre, sex, age at recruitment, time since transplantation and skin type by each HPV type, among Caucasian transplant patients without skin cancer from London and Oxford (N=425)

| **genus** | **species** |  | **All** |  | **CENTRE** | | |  | **SEX** | | |  | **AGE AT RECRUITMENT (years)** | | | |  | **TIME SINCE TRANSPLANTATION (years)** | | | |  | **SKIN TYPE** | | |
| --- | --- | --- | --- | --- | --- | --- | --- | --- | --- | --- | --- | --- | --- | --- | --- | --- | --- | --- | --- | --- | --- | --- | --- | --- | --- |
|  |  |  |  |  | London | Oxford |  |  | Male | Female |  |  | <45 | 45-59 | ≥ 60 |  |  | <5 | 5 to 9 | ≥10 |  |  | I/II | III/IV |  |
|  |  | **type** | **N=425 % POS** |  | **N=243 % POS** | **N=182 % POS** | ***P-value*¹** |  | **N=260 % POS** | **N=165 % POS** | ***P-value*²** |  | **N=187 % POS** | **N=158 % POS** | **N=80 % POS** | ***P-value trend*²** |  | **N=150 % POS** | **N=113 % POS** | **N=162 % POS** | ***P-value trend* ²** |  | **N=133 % POS** | **N=273 % POS** | ***P-value*²** |
| **alpha** | *1* | **3** | **8** |  | 9 | 8 | **0.9** |  | 8 | 9 | **0.7** |  | 9 | 8 | 8 | **0.6** |  | 10 | 7 | 8 | **0.5** |  | 6 | 10 | **0.2** |
|  | *4* | **2** | **14** |  | 17 | 11 | **0.09** |  | 13 | 16 | **0.5** |  | 18 | 12 | 11 | **0.1** |  | 11 | 18 | 15 | **0.5** |  | 14 | 14 | **1.0** |
|  |  | **27** | **17** |  | 21 | 12 | **0.01** |  | 15 | 21 | **0.1** |  | 24 | 12 | 13 | **0.01** |  | 14 | 21 | 17 | **0.6** |  | 20 | 16 | **0.6** |
|  | *8* | **7** | **8** |  | 9 | 7 | **0.4** |  | 7 | 12 | **0.1** |  | 11 | 6 | 6 | **0.09** |  | 6 | 11 | 9 | **0.4** |  | 8 | 8 | **0.7** |
|  | *9* | **16** | **16** |  | 16 | 15 | **0.6** |  | 10 | 25 | **<0.001** |  | 22 | 11 | 10 | **<0.001** |  | 16 | 15 | 16 | **0.9** |  | 14 | 16 | **0.3** |
|  | *10* | **6** | **30** |  | 33 | 26 | **0.1** |  | 29 | 32 | **0.5** |  | 36 | 28 | 20 | **0.01** |  | 31 | 24 | 33 | **0.8** |  | 29 | 31 | **0.4** |
|  |  | **13** | **10** |  | 13 | 5 | **0.01** |  | 8 | 13 | **0.04** |  | 16 | 4 | 8 | **<0.001** |  | 9 | 10 | 11 | **0.7** |  | 13 | 9 | **0.6** |
| **beta** | *1* | **5** | **9** |  | 11 | 7 | **0.2** |  | 10 | 7 | **0.3** |  | 10 | 9 | 8 | **0.5** |  | 5 | 11 | 12 | **0.06** |  | 9 | 9 | **0,9** |
|  |  | **8** | **21** |  | 24 | 18 | **0.2** |  | 21 | 22 | **0.9** |  | 26 | 18 | 18 | **0.06** |  | 16 | 24 | 25 | **0.1** |  | 21 | 21 | **0.9** |
|  |  | **20** | **14** |  | 16 | 12 | **0.3** |  | 15 | 13 | **0.4** |  | 15 | 12 | 18 | **0.8** |  | 11 | 14 | 18 | **0.1** |  | 12 | 15 | **0.4** |
|  |  | **24** | **11** |  | 11 | 12 | **0.8** |  | 11 | 12 | **0.9** |  | 13 | 8 | 13 | **0.7** |  | 8 | 16 | 10 | **0.5** |  | 9 | 11 | **0.5** |
|  |  | **36** | **12** |  | 15 | 8 | **0.03** |  | 11 | 13 | **0.7** |  | 13 | 10 | 13 | **0.8** |  | 8 | 12 | 15 | **0.1** |  | 10 | 11 | **0.5** |
|  |  | **93** | **3** |  | 5 | 1 | **0.0** |  | 2 | 4 | **0.5** |  | 2 | 3 | 4 | **0.5** |  | 2 | 3 | 4 | **0.5** |  | 3 | 3 | **0.8** |
|  | *2* | **9** | **15** |  | 19 | 11 | **0.03** |  | 15 | 15 | **1.0** |  | 17 | 15 | 14 | **0.6** |  | 13 | 19 | 15 | **0.7** |  | 14 | 15 | **0.5** |
|  |  | **15** | **27** |  | 26 | 29 | **0.5** |  | 27 | 28 | **0.9** |  | 27 | 31 | 20 | **0.5** |  | 18 | 40 | 27 | **0.07** |  | 23 | 29 | **0.4** |
|  |  | **17** | **24** |  | 25 | 21 | **0.3** |  | 21 | 27 | **0.2** |  | 24 | 23 | 24 | **0.9** |  | 19 | 34 | 21 | **0.8** |  | 21 | 25 | **0.3** |
|  |  | **23** | **10** |  | 12 | 7 | **0.07** |  | 9 | 10 | **0.8** |  | 9 | 9 | 11 | **0.6** |  | 5 | 13 | 11 | **0.1** |  | 8 | 11 | **0.3** |
|  |  | **38** | **22** |  | 23 | 21 | **0.8** |  | 22 | 23 | **0.9** |  | 22 | 23 | 21 | **0.9** |  | 14 | 30 | 25 | **0.03** |  | 19 | 24 | **0.3** |
|  | *3* | **49** | **20** |  | 19 | 21 | **0.5** |  | 18 | 22 | **0.4** |  | 22 | 18 | 18 | **0.2** |  | 14 | 21 | 24 | **0.02** |  | 20 | 19 | **1.0** |
|  |  | **75** | **12** |  | 14 | 9 | **0.2** |  | 12 | 12 | **0.9** |  | 12 | 12 | 11 | **1.0** |  | 7 | 14 | 15 | **0.03** |  | 9 | 13 | **0.2** |
|  |  | **76** | **10** |  | 12 | 7 | **0.1** |  | 9 | 11 | **0.6** |  | 9 | 11 | 9 | **0.9** |  | 5 | 12 | 13 | **0.03** |  | 8 | 11 | **0.3** |
|  | *4* | **92** | **12** |  | 15 | 8 | **0.04** |  | 12 | 13 | **0.8** |  | 12 | 13 | 11 | **1.0** |  | 8 | 13 | 15 | **0.07** |  | 14 | 12 | **0.8** |
|  | *5* | **96** | **15** |  | 15 | 14 | **0.9** |  | 13 | 16 | **0.5** |  | 13 | 16 | 15 | **0.6** |  | 9 | 20 | 16 | **0.1** |  | 12 | 16 | **0.4** |
| **gamma** | *1* | **4** | **25** |  | 27 | 23 | **0.5** |  | 27 | 23 | **0.4** |  | 28 | 27 | 16 | **0.09** |  | 17 | 30 | 30 | **0.01** |  | 23 | 26 | **0.6** |
|  |  | **65** | **28** |  | 30 | 25 | **0.2** |  | 27 | 30 | **0.4** |  | 35 | 25 | 19 | **<0.001** |  | 22 | 34 | 30 | **0.2** |  | 25 | 28 | **0.3** |
|  |  | **95** | **21** |  | 22 | 20 | **0.6** |  | 19 | 25 | **0.2** |  | 24 | 16 | 25 | **0.7** |  | 17 | 25 | 23 | **0.3** |  | 20 | 22 | **0.6** |
|  | *2* | **48** | **16** |  | 16 | 15 | **0.7** |  | 15 | 16 | **0.7** |  | 19 | 13 | 14 | **0.2** |  | 13 | 21 | 14 | **1.0** |  | 15 | 15 | **0.9** |
|  | *3* | **50** | **8** |  | 8 | 7 | **0.8** |  | 6 | 10 | **0.07** |  | 8 | 9 | 4 | **0.3** |  | 7 | 7 | 8 | **0.8** |  | 5 | 9 | **0.1** |
|  | *4* | **60** | **4** |  | 5 | 4 | **0.6** |  | 3 | 7 | **0.08** |  | 4 | 6 | 4 | **0.8** |  | 4 | 5 | 4 | **1.0** |  | 4 | 4 | **0.7** |
| **nu** | *1* | **41** | **11** |  | 13 | 8 | **0.06** |  | 11 | 11 | **1.0** |  | 11 | 11 | 11 | **0.9** |  | 11 | 11 | 10 | **0.7** |  | 12 | 10 | **0.8** |
| **mu** | *1* | **1** | **29** |  | 33 | 24 | **0.04** |  | 26 | 34 | **0.1** |  | 31 | 30 | 23 | **0.2** |  | 25 | 27 | 35 | **0.1** |  | 31 | 29 | **0.9** |
|  | *2* | **63** | **23** |  | 28 | 17 | **0.01** |  | 21 | 27 | **0.2** |  | 27 | 20 | 20 | **0.1** |  | 16 | 28 | 26 | **0.07** |  | 24 | 23 | **0.8** |
| **ND** |  | **101** | **7** |  | 10 | 3 | **<0.001** |  | 6 | 9 | **0.2** |  | 6 | 6 | 10 | **0.4** |  | 6 | 7 | 8 | **0.8** |  | 7 | 8 | **0.4** |
|  |  | **103** | **6** |  | 7 | 4 | **0.2** |  | 6 | 6 | **1.0** |  | 6 | 8 | 4 | **0.8** |  | 4 | 8 | 7 | **0.3** |  | 7 | 6 | **0.8** |

HPV: Human papillomavirus; POS: number seropositive patients; N: number; ND: Not defined. Skin type was defined as: (I) `rarely tans, usually burns’ (II) `usually tans, can burn’ (III) `always tans, rarely burns’ (IV) `always tans, never burns’. Some figures do not add up to 425 due to missing data. ¹ Analyses were based on unconditional logistic regression adjusted for sex, time since transplantation and age at recruitment. ² Analyses were based on conditional on centre logistic regression adjusted for sex, time since transplantation and age at recruitment. P-values are based on tests for heterogeneity calculated by likelihood ratio tests and tests for trend were obtained by treating categorical variable as a continuous variable in the model.
